# Postbariatric EArly discharge Controlled by Healthdot (PEACH) trial: study protocol for a preference-based randomized trial

**DOI:** 10.1186/s13063-022-06001-9

**Published:** 2022-01-21

**Authors:** Jai Scheerhoorn, Lisa van Ede, Misha D. P. Luyer, Marc P. Buise, R. Arthur Bouwman, Simon W. Nienhuijs

**Affiliations:** 1 Department of Surgery, Catharina Hospital, Eindhoven, The Netherlands; 2 Department of Anesthesiology, Catharina Hospital, Eindhoven, The Netherlands; 3 Department of Electrical Engineering, Eindhoven University of Technology, Eindhoven, The Netherlands

**Keywords:** Bariatric surgery, Telemonitoring, Outpatient surgery, Home monitoring, Patient preference randomized trial, Study protocol

## Abstract

**Introduction:**

Performing bariatric surgery in a daycare setting has a potential reduction in hospital costs and increase in patients’ satisfaction. Although the feasibility and safety of such care pathway has already been proven, its implementation is hampered by concerns about timely detection of short-term complications. This study is designed to evaluate a combined outcome measurement in outpatient bariatric surgery supplemented by a novel wireless remote monitoring system versus current standard of care.

**Methods and analysis:**

A total of 200 patients with multidisciplinary team approval for primary bariatric surgery will be assigned based on their preference to one of two postoperative trajectories: (1) standard of in-hospital care with discharge on the first postoperative day or (2) same day discharge with ongoing telemonitoring up to 7 days after surgery. The device (Healthdot R Philips) transfers heart rate, respiration rate, activity, and body posture of the patient continuously by LoRaWan network to our hospital’s dashboard (Philips Guardian). The primary outcome is a composite outcome measure within 30 days postoperative based on mortality, mild and severe complications, readmission, and prolonged length-of-stay. Secondary outcomes include patients’ satisfaction and data handling dimensions.

**Trial registration:**

ClinicalTrials.govNCT04754893, Registered on 12 February 2021**.**

## Background

Bariatric surgery has proven its worth for the treatment of obesity and is considered to be the most effective long-term treatment option [1–3]. Implementation of Enhanced Recovery After Bariatric Surgery (ERABS) reduced the length of hospital stay (LOS) without significant influence on overall morbidity [4, 5]. Furthermore, it reduced risk of hospital-acquired infections, improved accessibility to bariatric surgery, improved patient and health professional satisfaction, and increased cost-effectiveness [6, 7]. Current standard of care in The Netherlands is to discharge patients 1 day postoperatively [8].

Literature has proven the safety of performing outpatient bariatric surgery such as laparoscopic gastric sleeve gastrectomy (SG) and Roux-en-Y gastric bypass (RYGB) [4, 9–14]. Despite being proven to be safe, the implementation of outpatient bariatric surgery is not yet widespread. Underlying reason relates to the concern that short-term complications such as bleeding may not be timely be detected [15, 16]. Most often these complications first express themselves by a change in vital parameters such as tachycardia. A wireless remote monitoring system would allow monitoring of vital signs in patients that have been discharged shortly following surgery and facilitate detection of early postoperative complications in a home environment. Moreover, telemonitoring could also be of added value to detect late-term complications which occur in a home situation.

Even though many wireless monitoring devices are available, most are still in clinical validation and feasibility testing phases [17], and bear the disadvantage that vital signs data have to be uploaded via a datahub. Experience from previous studies learnt that home monitoring was hampered in the home situation by difficulties with regard to connectivity issues and ease of use leading to incomplete data acquisition. New clinically tested and validated devices have become available, such as Healthdot (Philips Electronic Nederland BV), that continuously collect vital signs and supports automated data transmission towards a vital signs dashboard in the hospital and as such increasing ease of use and patient comfort [18]. Using this device it seems possible to discharge patients on the day of surgery without compromising the early detection of short-term complications.

The primary objective of this patient preference-based randomized trial is to that implementation of ambulatory bariatric surgery combined with remote monitoring up to 7 days after surgery using a wireless unobtrusive continuous monitoring device with automated data transmission, is not inferior to the current standard postoperative care, while maintaining patient comfort and satisfaction.

## Methods and analysis

### Study design

**Fig. 1:**
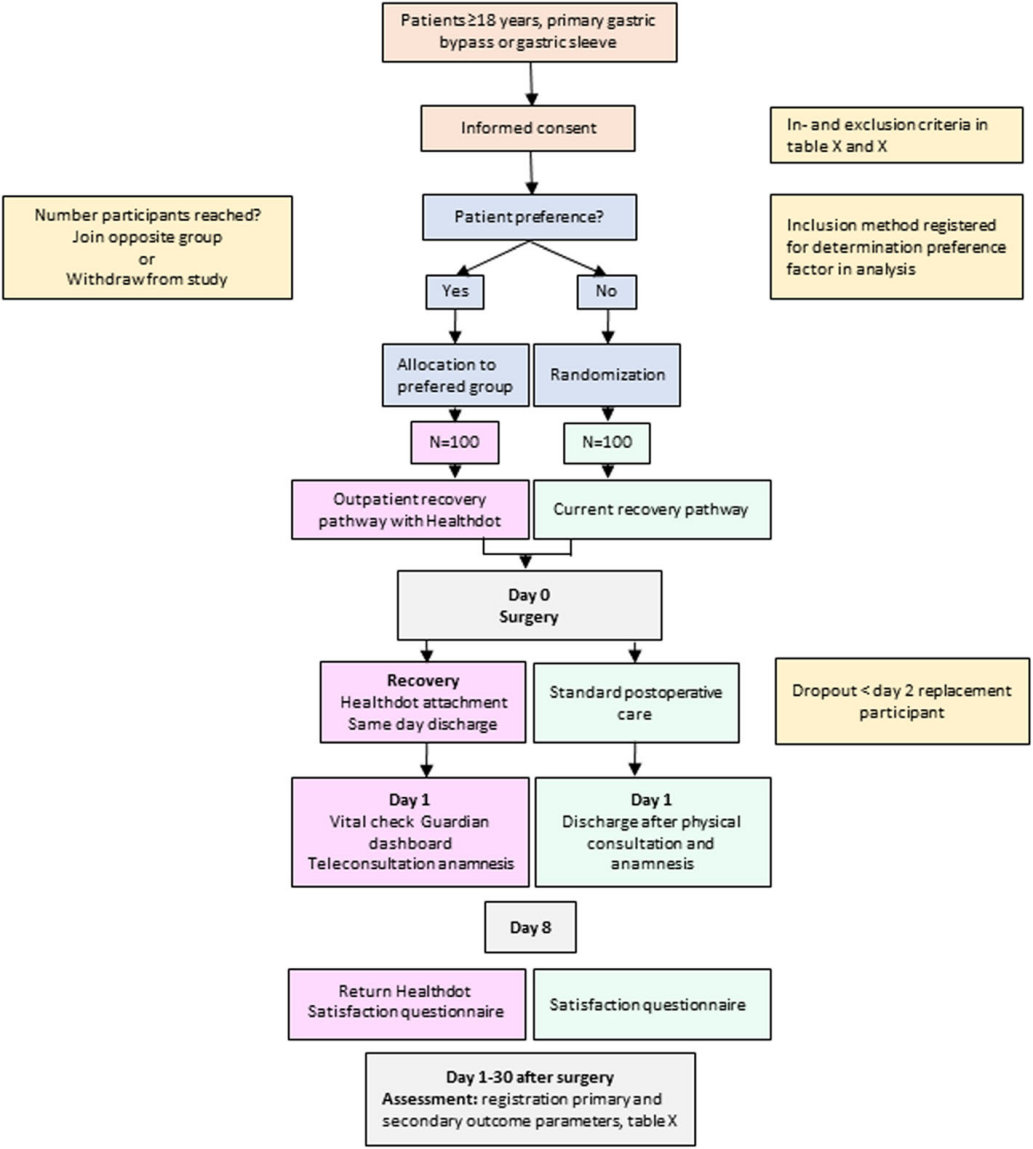
Outline of the study pathway

This single-center preference-based randomized trial in a tertiary hospital in the Netherlands is designed to compare the outcomes of two different recovery pathways after standard care bariatric surgery. Figure 1 shows an outline of the study pathway.

Primary outcome is a combined measurement of mortality, severe postoperative complications (Clavien-Dindo IIIb or higher), mild complications (Clavien-Dindo II and IIIa), hospital readmission, or prolonged length of stay, all within 30 days after surgery. Secondary outcomes include patients’ satisfaction, data handling dimensions, post-operative analgesia, and cost-effectiveness.

Randomized controlled trials (RCT) are suggested to provide the most reliable evidence for treatment efficacy [19]; however, treatment preferences can decrease the generalizability of the results and reduce its external validity. Nevertheless, recent literature has shown the added value of including patient preference in medical research [20–22]. Throughout the years, various alternative designs have been suggested to diminish the influence of patients’ preference on validity. One of these approaches is the partially randomized patient preference trial (RPPT). Recently, a systematic review has shown patients preference did not influence the primary outcome despite of a substantial proportion of a specific patient group refusing randomization [19]. Therefore, compared to RCTs, a RPPT can increase external validity without compromising the internal validity. In current daily clinical practice, patient preference has a significant impact on decisions made by clinicians regarding their treatment or treatment pathway. Hence we believe that implementation of this new recovery pathway comes to its best when mimicking daily practice as much as possible. Therefore, in our opinion, an RPPT is best suitable to investigate our endpoints.

### Study population

The patient is eligible to participate in the study if they are planned for a primary laparoscopic gastric bypass or gastric sleeve. Inclusion and exclusion criteria are mentioned in Tables 1 and 2, respectively. Any patient referred to our center is screened by a multidisciplinary team for eligibility for bariatric surgery. They are asked to fill out screening questionnaires and receive presentation and interviews by a dietician, obesity nurse, psychologist, and physiotherapist and receive extensive blood tests. The combined findings of this screening are discussed in a multidisciplinary session in which the surgeon is also involved. When approved, patients visit the bariatric surgeon to determine which bariatric procedure is deemed most suitable. After informed consent has been given, it will be determined whether the patient prefers the current standard of care or the outpatient recovery pathway. Patients who choose the outpatient pathway have to make sure there is someone with them the night after the surgery who is able to contact the hospital when questions arise and need to be able to mobilize help in case of a medical emergency. The factor of preference will be included in the analysis. If the preferred group is full, the patient can choose either to participate in the other group or withdraw from the study. The patients without preference are randomized in a 1:1 ratio. Simple randomization is used, meaning every individual has an equal chance to be enrolled in one of the two groups. Allocation is into one of two groups: (1) outpatient group (*n* = 100) or (2) current standard of care group (*n* = 100). Patients will be randomly assigned through a computer-generated randomization program. This program will take into account group size to end up with an equal number. Allocation concealment for patients without preference will be ensured, as the allocation of patients is generated after the patient signs the informed consent form and thereby included in the study

**Table 1:** Inclusion criteria

Willing and able to sign informed consent form	
Able to understand instructions	
An adult person must be present at the same location as the patient during the first night following surgery who is able to mobilize help or seek medical care if necessary	
In possession of a telephone on which patient can be reached for the duration of participation (days 1–8)

**Table 2:** Exclusion criteria

Patients of psychiatric wards, inmates or prisons, or other state institutions	
Investigator or any other team member involved directly or indirectly in the conduct of the clinical study	
Any skin condition, for example, prior rash, discoloration, scars, or open wounds at the area where the Healthdot needs to be placed	
Known allergy for the tissue adhesive used in the Healthdot (white band-aid)	
Use of topical that is known to influence the skin at the test area (such as medical and non-medical creams or lotions)	
Patient with active implantables such as Implantable Cardioverter Defibrillator (ICD) and pacemaker	
Left lower rib (place where Healthdot will be applied) is involved in the area of surgery, area of disinfection, or area where bandages are needed.	
Expected participation less than 8 days	

Patients who change their minds (change preference) before surgery can switch to the other study group. If a patient changes his/her preference between surgery and discharge this will be noted as a failure to implement the new protocol. In case same day discharge is not deemed feasible for surgical and/or medical reasons at the discretion of the attending surgeon or anesthesiologist, the patient will be noted as failure as well. Any change in patient preference/study group will be registered. Any other situation will be considered as a dropout (Table 3).

**Table 3:** Dropout criteria

Patients who are unwilling to further participate in the study	
Patients who did not undergo surgery (for example new onset of disease which makes the patient unsuitable for surgery)	
Concomitant new onset of a disease influencing the outcome	

### Outcome measures

The primary outcome of the study is the absence of the following within 30 days after primary surgery.

1. Mortality
2. Severe postoperative complications (Clavien-Dindo IIIb or higher)
3. Readmission (at least one night in hospital)
4. Mild complications (Clavien-Dindo II and IIIa)
5. Prolonged length of stay (3 days or more in hospital)

Secondary outcome measures are shown in Table 4.

**Table 4:** Secondary outcome measures

Patient satisfaction > 6 points (on a scale of 1–10)	
Percentage of patients recruited for the outpatient recovery after standard bariatric surgery group	
Percentage of patients with full adherence to protocol and randomization	
Percentage of missing data	
Percentage of false positive of the positive notifications from the Healthdot system	
Total number of false positive of the positive notifications from the Healthdot system	
Total number of false negative notifications from the Healthdot system	
The outcome of patients who choose HD versus patients who were randomized to HD on a combined outcome measure as defined for the primary endpoint.	
To evaluate the outcome of patients who choose HD versus patients who were randomized to HD on a combined outcome measure as defined for the primary endpoint.	
Number of adverse events in both groups	
Amount of pain medication taken on the day of surgery in both groups	
Percentage of clinical decisions made based on the Healthdot system, that changed when information from a telephone consultation was also included.	
Patient and health professional satisfaction (on a scale from 1 to 10) for both the outpatient recovery after standard bariatric surgery group as the current recovery path group	
The costs involved with outpatient recovery after standard bariatric surgery supported by Healthdot and the cost involved with the current recovery path, in the period up to 7 days after surgery.	

### Sample size calculation

Sample size calculation was based on a previous study in the Netherlands in which the primary outcome measure was used [23]. In this study, the average outcome across bariatric surgery patients in the Netherlands was 88.7%. In current practice in the Catharina Hospital, Eindhoven, 95% of all bariatric surgery patients meet this combined outcome measure. This helps to derive a non-inferiority margin of around 7%. A *p* value of 0.05 will be considered statistically significant.

Sample size calculations were performed using PS: Power and Sample Size Calculation software (v3.1.2, Vanderbilt University). With a power of 0.80, an *α* of 0.05, a non-inferiority margin of 0.07, and an expected proportion of 0.96, this resulted in a sample size of 97 patients per group using an uncorrected chi-squared statistic.

The expected drop-out rate is 10% (20 patients in this sample). Patients dropping out before study start or during the first 2 days of the study will be replaced until the final 200 patients are included and have participated more than the 2 days defined for replacement.

The interim analysis will take place when data from 50 patients has been collected. The data of this study will not be monitored during the study. The study will be monitored. Monitor visits will occur after; 15 patients have been included, 50 patients have completed follow-up, and once all patients have completed follow-up.

### Recruitment

Participant recruitment will occur in our outpatient clinic. Every patient approved for bariatric surgery will be approached to participate by one of the study members. With around 900 bariatric procedures a year in the Catharina Hospital, the supply of eligible patients is large enough. Given the design of our study in which patients can decide in which group to participate, we believe that the participation rate will be high.

### The Healthdot system

**Fig. 2:**
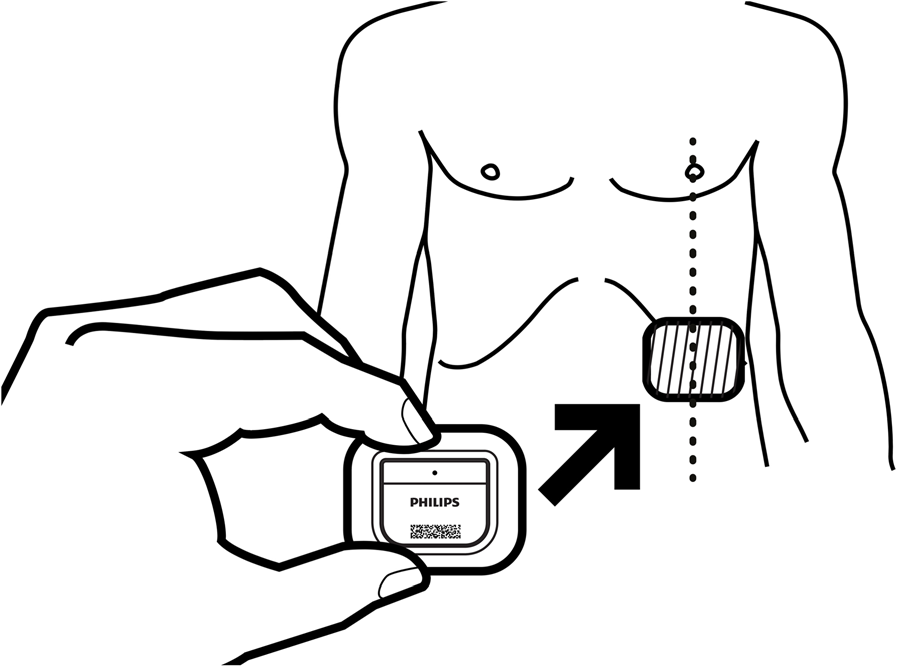
Schematic view of the Healthdot. Reprinted from Philips Electronic Nederland BV under a CC BY license, with permission from Philips Electronic Nederland BV, original copyright 2020

The Healthdot system (Philips Electronic Nederland BV) consists of a wearable data logger that transmits data to be visualized real-time on our hospital dashboard after pairing the Healthdot to the patient. The data logger device consists of an adhesive layer, electronics, and a battery (Fig. 2). The general operating principle is based on the continuous collection and periodic transmission of heart rate, respiratory rate, activity, and posture by means of processing of accelerometer signals. Every 5 min, the mean of the data is transmitted from the Healthdot device to a Cloud Server (Health Suite Digital Platform, HSDP, Philips electronics Nederland bv) using the KPN LoRaWan network, as a part of the Philips Guardian System.

### The Guardian Dashboard

Data of the parameters (heart rate, respiration rate, activity, and body posture) will be available to view in the in hospital Guardian Dashboard (Philips Intellivue Guardian: release E, Enterprise release 13.1). Once logged into the system, the vitals are presented as trend figures. Events with a heart rate or respiration rate above a pre-defined threshold will be flagged and a warning score will be calculated. There will be no alarm notifications when not logged into the system.

Literature has shown that the heart rate of patients after bariatric surgery complicated by either anastomotic leakage or postoperative bleeding is > 120 beats per minute (bpm) in respectively 87.5% vs 35% and > 100–120 bpm in 12,5% and 60% of the patients [24–26]. Based on literature and own experience of the medical team, the threshold was set at a heart rate above 110 beats per minute (bpm) and a respiration rate above 20 with a reassurance period of 15 min.

## Intervention

### Start of the study

Patients who have chosen to undergo outpatient surgery will be scheduled for surgery on the earliest available time-slots in the morning. The Healthdot will be applied mid-clavicular on the lowest left rib of the chest as the patient is in the recovery room. The Healthdot can be removed by the patient themselves in case of emergency with a remover tissue which will be handed out at discharge. The postoperative care will be arranged in such a way that same day discharge is possible for this group of patients. Patients who have chosen the regular treatment will receive care in accordance with the current protocol.

### Discharge from hospital

At the end of the day, patients in the outpatient surgery group will be discharged from the hospital on the discretion of a nurse under supervision of a physician, wearing the Healthdot. Patients will only be discharged if they meet the following criteria: pain should be adequately controlled with oral medication, there should be no nausea or vomiting, the patient is mobilizing and the patient is willing to be discharged. Furthermore, clinical findings should show no fever, a heart rate ≤110 bpm, and an adequate oral intake. Laboratory findings should show a no hemoglobin decrease of more than 2 mmol/l when compared to the preoperative value. The physician will be able to view the parameters on the Guardian dashboard when measured by Healthdot at all times when logged into the system, including the flag marks and warning scores. A teleconsultation will be scheduled for the following morning (day 2). Before the teleconsultation, the vital signs in the dashboard will be interpreted by the physician who will then assess whether additional action is indicated. The decision based on Healthdot data is noted. After the teleconsultation, it is noted whether additional or different decisions are made about the treatment based on the additional information from the teleconsultation.

On days 3–8, the physician or a nurse will check the Guardian dashboard daily. The physician may decide to have a telephone consultation, e.g., when the Healthdot dashboard flags an event. If the physician determines that additional action is needed (e.g., based on the information on the dashboard, or when the patient calls), in the absence of an indication from the Healthdot, such an event will be viewed as a false negative. It will be retrospectively assessed, based on the patient medical record, if false negative events have occurred. For all events flagged in the Healthdot dashboard, the physician will record whether it was a false positive notification or a true positive notification.

Patients in the regular treatment group will be released from the hospital at the discretion of the physician the day after surgery. For these patients, no appointments outside of the regular protocol are planned.

### Follow-up

Patients who received the Healthdot will remove this themselves and are requested to send the device back to the hospital on day 8. At hospital discharge, the patient will be handed out a return envelope including the return instructions. The envelope also includes a patient/healthcare satisfaction questionnaire they are requested to fill in and send along with the device. The presence or absence of readmission within 30 days will be observed from the patient record and collected in a anonymized database. Health care professionals will also be requested to fill in a short satisfaction questionnaire after the end of the study.

**Fig. 3:**
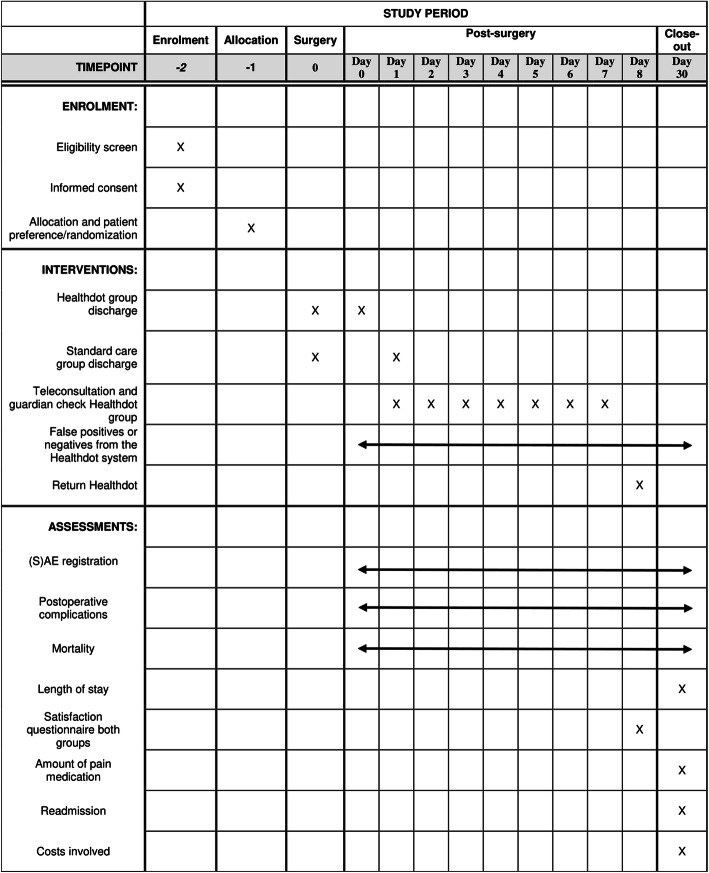
Schedule of this trial

The schedule of this trial is shown in Fig. 3.

### Discussion

In this study we aim to demonstrate bariatric surgery as outpatient surgery combined with remote monitoring is non-inferior to the current standard postoperative care, while patient comfort and satisfaction is maintained.

Healthcare has developed rapidly over the last decades. The implementation of ERAS protocols in bariatric surgery has led to improved outcomes and has shortened length of stay to the point where bariatric surgery is being performed as outpatient procedures [4, 5, 8]. Recent technological development has led to novel devices that can wirelessly monitor the vital parameters of a patient in the hospital as well as at home. Therefore, in theory, patients no longer need to stay admitted to be able to detect post-operative complications. This is the first patient preference-based randomized trial in which bariatric surgery will be performed as an outpatient procedure and the patients’ vital parameters will be monitored at home using a novel device. There are some studies that use the surgical patient as a research population. However, these are usually studies which take place in-hospital or in which the patient has been in the hospital for several days between surgery and discharge [27–30]. Therefore, these studies cannot be directly compared with this study.

Some limitations need to be addressed as well. Being a patient preference-based randomized trail instead of a randomized control trial (RCT), the patient being able to choose his or her treatment can lead to bias. For example, it is possible that young patients are more inclined to participate in the outpatient treatment group then older patients. This can impact outcomes and will need to be taken into consideration during statistical analysis. Although, as mentioned previously, recent studies have shown that compared to RCTs, a RPPT can increase external validity without compromising the internal validity [19].

Using a relatively new medical device (Healthdot) in this study can also influence results. The impact of false- positives or negatives from the Healthdot system on the outcomes is unknown. The notification thresholds used in this study are based on a combination of literature and own experience but are not yet defined on analysis. This study will provide insight in data whereby a protocol like this can be adjusted and specified. Furthermore, the study requires a new treatment pathway to be implemented in daily clinical practice which could take time and effort from nurses and other health workers.

This study will give insights into the safety of outpatient bariatric surgery when combined with telemonitoring in a large single-center clinical trial. It will also shed light on patient satisfaction when performing bariatric surgery on an outpatient basis compared to the current standard and, furthermore, can answer the question if costs can be saved by implementing this new pathway.

## Data Availability

All investigators will have access to the datasets. The project datasets will be hosted on the hospital’s secure network and all datasets will be password protected. Anonymized data will be available for Philips Electronics Nederland B.V. for development purposes.

## Declarations

### Protocol amendments

In the case of a necessary protocol amendment, the amendment will be submitted to the ethics committee.
